# Identification of CD38, CD97, and CD278 on the HIV surface using a novel flow virometry screening assay

**DOI:** 10.1038/s41598-023-50365-0

**Published:** 2023-12-27

**Authors:** Jonathan Burnie, Claire Fernandes, Deepa Chaphekar, Danlan Wei, Shubeen Ahmed, Arvin Tejnarine Persaud, Nawrah Khader, Claudia Cicala, James Arthos, Vera A. Tang, Christina Guzzo

**Affiliations:** 1 Department of Biological Sciences, University of Toronto Scarborough, 1265 Military Trail, Toronto, ON Canada; 2 Department of Cell and Systems Biology, University of Toronto, 25 Harbord Street, Toronto, ON Canada; 3 Laboratory of Immunoregulation, National Institute of Allergy and Infectious Diseases, National Institutes of Health, Bethesda, MD USA; 4 Flow Cytometry and Virometry Core Facility, Department of Biochemistry, Microbiology, and Immunology, Faculty of Medicine, University of Ottawa, 451 Smyth Road, Ottawa, ON Canada

**Keywords:** Microbiology techniques, Retrovirus, Virus-host interactions

## Abstract

While numerous cellular proteins in the HIV envelope are known to alter virus infection, methodology to rapidly phenotype the virion surface in a high throughput, single virion manner is lacking. Thus, many human proteins may exist on the virion surface that remain undescribed. Herein, we developed a novel flow virometry screening assay to discover new proteins on the surface of HIV particles. By screening a CD4^+^ T cell line and its progeny virions, along with four HIV isolates produced in primary cells, we discovered 59 new candidate proteins in the HIV envelope that were consistently detected across diverse HIV isolates. Among these discoveries, CD38, CD97, and CD278 were consistently present at high levels on virions when using orthogonal techniques to corroborate flow virometry results. This study yields new discoveries about virus biology and demonstrates the utility and feasibility of a novel flow virometry assay to phenotype individual virions.

## Introduction

Viral envelope glycoproteins are paramount in initiating infection and directing virus tropism. While the importance of viral glycoproteins has been well appreciated, the study of cellular proteins which become incorporated into viral particles is also highly relevant to infection (reviewed in ^1^). For example, cellular proteins in the membrane of enveloped viruses have shown impacts on viral infection through altering viral attachment, neutralization sensitivity and infectivity^2–7^. This is largely due to the fact that some cellular proteins can maintain their basic biology when present on virions and can impart new functionality to virions upon incorporation^8,9^. While this has been shown for cellular proteins on vaccinia virus, SIV and HIV (reviewed in ^1,9,10^), the majority of this work has been performed on HIV, which is the focus of this study. Recent reports identifying new proteins on HIV virions^6,11,12^ demonstrate the continued potential for ongoing discovery of novel proteins on HIV. One reason for this may be that the identification of HIV-incorporated proteins has been largely dependent on just a few traditional techniques, and therefore is subject to the specific limitations and caveats for detection with those methods.

Mass spectrometry is an example of a powerful technique which can be used to determine the broad range of proteins associated with a virus preparation. The technique has been used to provide comprehensive lists of cellular proteins found associated with virus preparations^13–17^. Despite this, the large amount of data provided from these assays presents challenges in determining which hits are biologically relevant to further characterize. Similarly, since mass spectrometry evaluates total protein content, it does not discriminate between proteins that are on the virion surface versus the virion interior. This is a notable limitation because cellular proteins that become incorporated within virions (intravirion) are likely to play less of a role in viral attachment and entry, due to their internal localization. However, many cellular proteins which decorate the HIV surface have been shown to alter virus attachment, entry and homing^5,6,18–22^. Many of these surface proteins which maintain functional activity on virions were identified through antibody-based screens^23–25^. Antibody-based methodology may offer a specific advantage to identify functionally active proteins on virions, particularly if the antibody recognizes an epitope on a functionally active domain.

The majority of past antibody screens performed on HIV were small scale and involved immunogold labelling paired with electron microscopy (EM), or antibody-mediated virus capture assays^19,23–28^. While these techniques were fundamental in developing our understanding of virion-incorporated proteins, all techniques have their own limitations. For instance, the sample processing required for EM and the low-throughput nature of manual imaging makes this method of identification time intensive for protein discovery. Conversely, while virion capture techniques can be performed in a short time frame, they offer information on total virus preparations and lack the resolution generated from single virion analyses.

The emerging technique flow virometry (FV), also known as nanoscale flow cytometry, which applies flow cytometry to viruses, provides a way to overcome the limitations specific to low throughput or bulk analyses. Similar to flow cytometry, flow virometry can provide high throughput characterization of individual virions^29^. Due to the widespread use of flow cytometry, many commercially available flow-based cell screening assays exist. However, while cellular phenotyping through flow cytometry is commonly used, this area is rather limited for viruses. Performing large scale screening experiments probing virion surfaces for cellular proteins has the potential to identify new markers on the virus, which could be useful for improving our understanding of viral diseases. Notably, while large scale antibody screening by flow cytometry has been performed for many cell types, this method has not been applied for screening of viruses with single particle resolution.

Herein we adapted a commercially available, flow cytometry-based cell screening panel (BioLegend LEGENDScreen) to phenotype the surface of four different HIV-1 isolates using FV. Our study screened for over 360 cell surface antigens, showing that robust and consistent staining can be generated when using FV techniques to screen the surface of viruses. From our screening efforts, we identified 59 new candidate human proteins in the HIV envelope, in addition to corroborating the presence of many other well-established virion-incorporated proteins, such as ICAM-1, CD44 and HLA DR. Finally, we demonstrated the utility of our method to identify three novel virion-incorporated proteins (CD38, CD97, and CD278), by using the traditional method of virion capture to corroborate results from FV assays. These newly identified virion-incorporated proteins may influence virus biology and attachment to immune cells, and may also represent novel biomarkers of interest on virus particles, particularly for CD38, as expression of CD38 on CD4+ T cells has been previously established as a prognostic marker in HIV disease^30,31^.

## Results

### Adapting the LEGENDScreen kit for staining HIV virions

To generate a robust human protein profile of the HIV surface, we used the LEGENDScreen (BioLegend) cell screening kit and adapted it for use on virions with our unique flow virometry methods. We employed this kit since it contains 360 PE-labelled monoclonal antibodies against cell surface markers and the appropriate isotype controls for flow cytometry staining in a convenient 96 well plate format. Furthermore, the kit contains antibodies for many proteins known to be incorporated in HIV which could serve as robust assay controls. Thus, we anticipated it would allow for a broad range of cellular markers to be detected on the surface of HIV. While viruses produced in primary cells are the most physiologically relevant to study, we chose to initially optimize the LEGENDScreen for use on virions produced in a CD4^+^ T cell line. Our prior work has shown that viruses from cell lines are more suitable than viruses from peripheral blood mononuclear cells (PBMC) for flow virometry optimization experiments because they generate highly monodisperse and homogenous virus populations on dot plots^22^.

**Figure 1:**
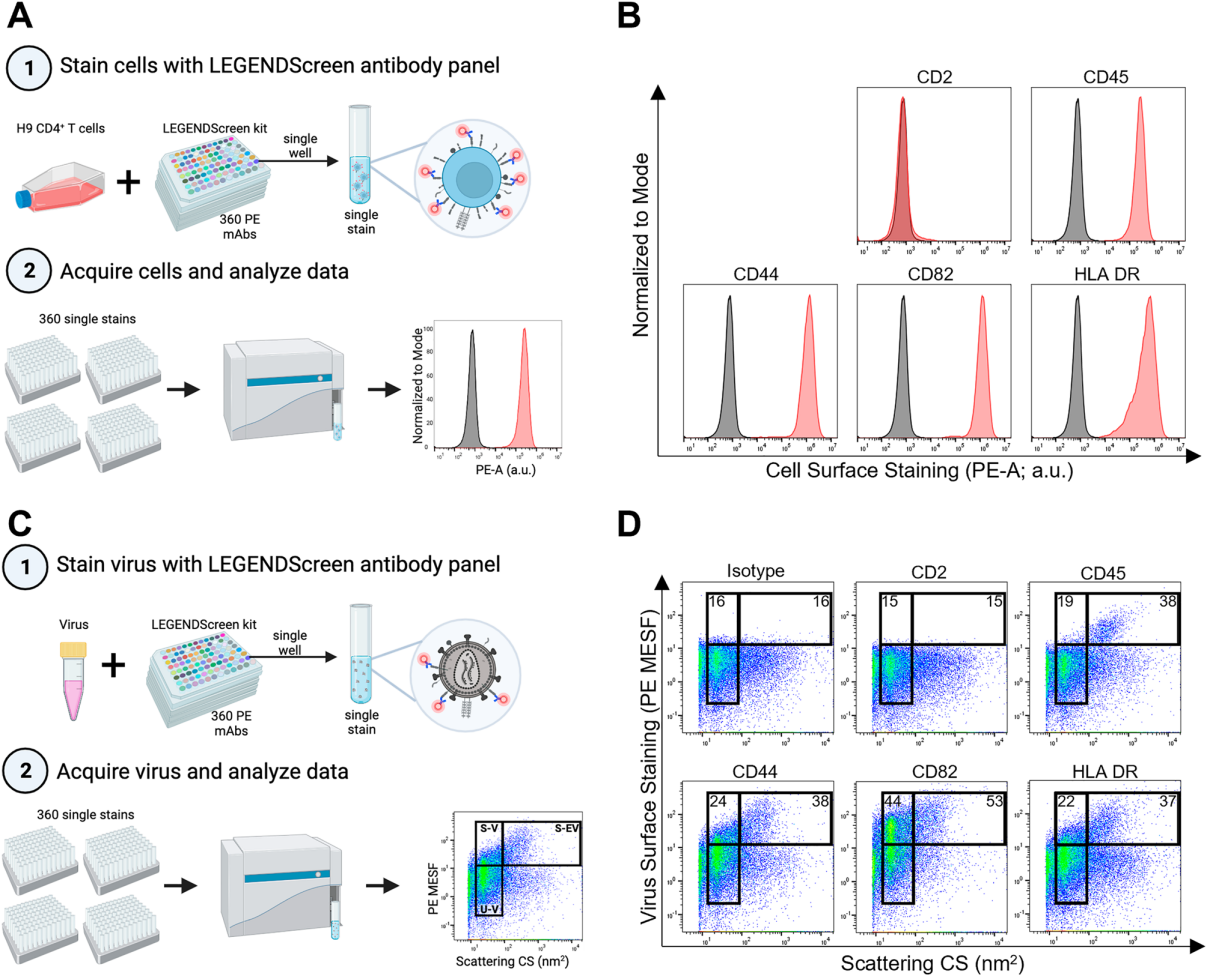
Adapting the LEGENDScreen kit for use on HIV_IIIB_ and its producer cells. (**A**) Schematic depicting the experimental workflow used for cell staining. Step 1 shows infected H9 cells stained with 360 unique PE-labelled antibodies provided in the LEGENDScreen kit, which consists of four 96-well plates containing a single PE-labelled mAb clone in each well (different clones depicted as different colors). Each well was harvested into a single tube, as a single stain to be acquired in step 2. Step 2 shows stained cells acquired on the CytoFLEX nanoscale cytometer by flow cytometry methods. Data are displayed as fluorescence histograms with isotype staining shown in black and specific protein staining shown in red. (**B**) A representative subset of cell surface staining from the LEGENDScreen. (**C**) Schematic depicting the experimental workflow used for virus staining. Step 1 shows virus (HIV_IIIB_) produced in H9 cells that was stained with the LEGENDScreen antibodies and then transferred to tubes and diluted in PBS before proceeding to step 2 for acquisition. Data are displayed in pseudocolour dot plots to allow for the visualization of virus and vesicle populations. The lower gate denotes unstained virus (U-V). The left and right upper gates display stained virus (S-V) and stained extracellular vesicles (S-EV), respectively. (**D**) A representative subset of virus surface staining from the LEGENDScreen, with mean PE MESF values depicted in the gates, as shown. Data from one screen performed on the H9 cells and their respective viruses are shown.

We produced the lab adapted isolate HIV_IIIB_ in the H9 CD4^+^ T cell line, since viruses from this cell type have been used previously to study virion-incorporated host proteins^19,23,26,32^. Before attempting to stain the viral stock, we first validated the LEGENDScreen assay on the H9 cells that were used to produce virus. This would allow for a complementary protein profile from both viral progeny and the virus producer cells to be generated. The H9 cells were stained with the full panel of antibodies in individual wells for each antibody tested and were acquired on the cytometer following the manufacturer’s instructions (Fig. 1A). As expected, we observed robust staining for common antigens on T cells, such as CD44, CD45, CD82 and HLA-DR (Fig. 1B). Similarly, the cell staining was also shown to be specific, and proteins which were expected to be absent on the cell line, such as CD2^33^, stained below background. A full list of the cell staining results for H9 cells is shown in Supplementary Table 1.

After validating the screening kit on H9 cells, we next stained the progeny virions from H9 cells using the same panel of LEGENDScreen antibodies (Fig. 1C). We chose to dilute the antibodies 100-fold from the recommended cellular concentrations, since this would allow for a final antibody staining concentration of ~ 0.5–2 µg/mL on virions. This range has been determined empirically by our lab and others to be appropriate for staining a variety of antigens on viruses^22,34–36^. We used pseudocolour dot plots to visualize our virus staining results since this data representation allows better visualization of particle heterogeneity. More specifically, in a given virus sample, both viruses and non-virus vesicles can be visualized based on their scattering properties when visualized with light scatter vs fluorescence. We utilized three different gates for analyses which are similar to what we have reported previously^34^: the lower gate contains unstained virus events, the upper left gate contains stained virus events (S-V), and the upper right gate contains stained extracellular vesicle events (S-EV, Fig. 1D). It is worth noting that while most of the events in our virus gates will belong to virions, one of the caveats of FV is that background instrument fluorescence and extracellular vesicles (EV) can also be present to some degree in all gates. Additional controls described throughout the manuscript will be used to address these considerations.

When assessing the virus staining from the screen, we compared the same subset of antigens stained on the surface of H9 cells to that on the surface of progeny virions. As expected, we saw minimal levels of positive staining when using the isotype control in both our virus gates (Fig. 1D; upper left) and EV gates (upper right). Appreciable levels of staining were observed in the virus gate (upper left) and vesicle gate (upper right) for molecules known to be present on the surface of viruses and vesicles, such as CD44, CD82 and HLA DR^24,37,38^. Although the levels of staining on viruses can appear modest compared to those seen on cells, the values shown here are in line with what we would expect on an HIV virion where the number of surface glycoproteins, including the viral spike protein, are typically present at very low levels^39–41^. Importantly, many antigens from the screen stained at a high mean fluorescence intensity, including a large number of positive control proteins that were previously established to be on HIV virions. All positive control proteins previously published in the literature (to our knowledge) are highlighted in Supplementary Tables 2 and 3, showing the PE MESF staining values with and without isotype staining subtracted, respectively. Furthermore, given the fact that many of the antigens targeted in the LEGENDScreen antibody panel are not specific to lymphocytes, the virus staining from the screen was shown to be highly specific, as there was no staining detected for any non-lymphocyte markers. As seen in Fig. 1, CD2 served as a good control for assay specificity, in which no staining was present on the virus, similar to what was seen with the producer cells (H9 cells). As an additional control, while we did observe high levels of CD45 staining on H9 cells (Fig. 1B), only negligible amounts of virus staining were detected with the anti-CD45 antibody (Fig. 1D; upper left gate). Contrarily, robust staining on larger ‘non-virus’ vesicles was observed (Fig. 1D; upper right gate), as expected based on previous literature^42–44^. Taken together, these results support using flow virometry screening assays to discover cellular proteins on the surface of HIV.

### Discovering human proteins on HIV virions produced in PBMC with a novel flow virometry screen

**Figure 2:**
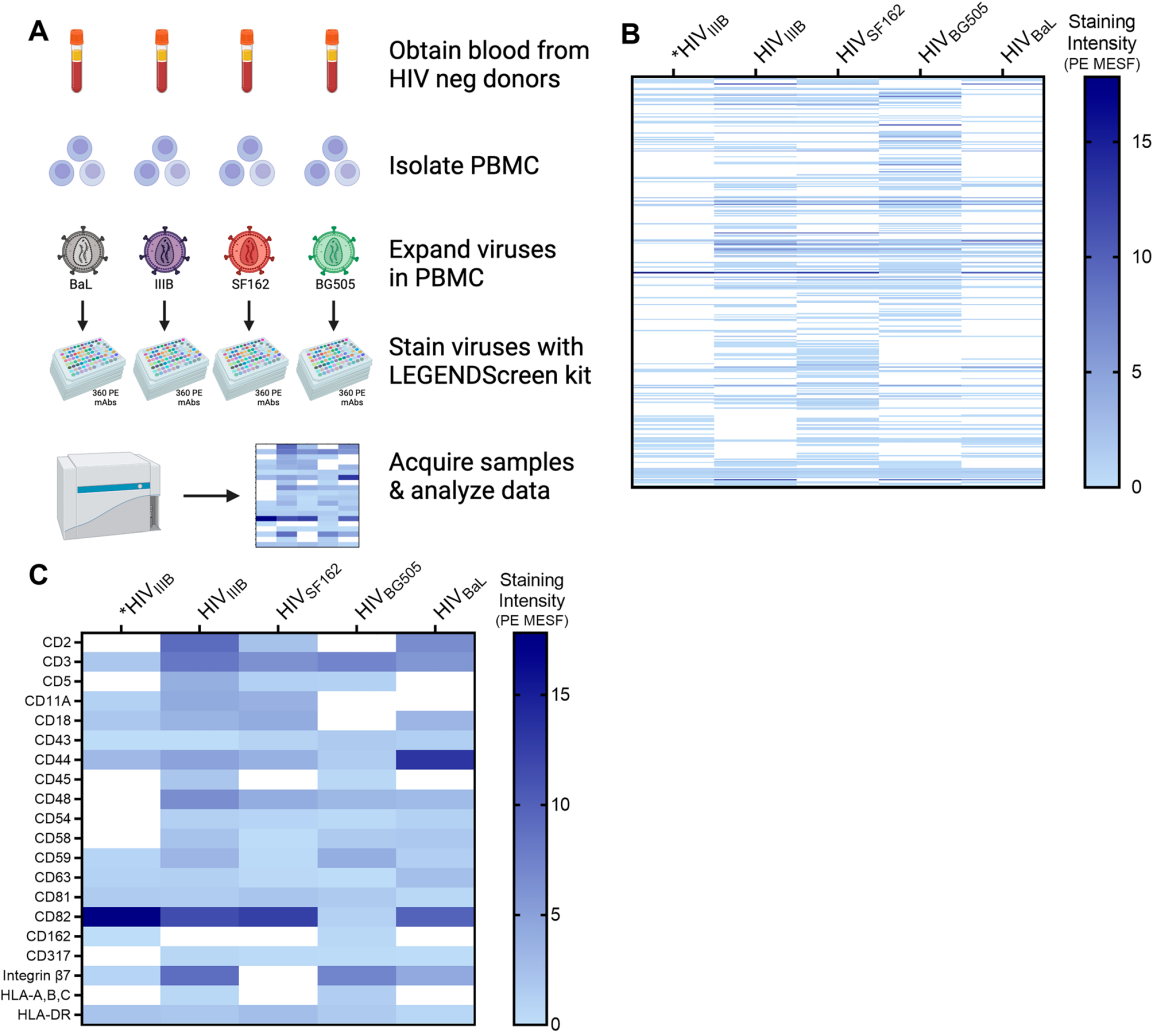
Screening viruses from primary cells using a novel flow virometry assay. (**A**) Schematic depicting the experimental workflow used for staining PBMC viruses. Blood samples from four uninfected donors were used to isolate PBMC. Four different HIV isolates (BaL, IIIB, SF162, BG505) were propagated in PBMC. Each viral stock was then stained individually with the full panel of LEGENDScreen antibodies before being diluted with PBS, and then acquired on the cytometer. Data are displayed in a heatmap with darker shades representing higher levels of staining. (**B**) Heat map displaying the mean fluorescence (PE MESF) generated from each of the stained PBMC virus isolates that were tested (columns 2–5). Each row represents a different antibody stain from the LEGENDScreen panel, and each column represents a different viral isolate. Staining intensities for proteins of interest were subtracted from the mean staining values generated from staining with matched isotype controls. Samples with staining below the isotype control are shown in white. *Staining intensities from HIV_IIIB_ produced in the H9 T cell line are shown in column one, whereas columns 2–5 show viral isolates propagated in PBMC. (**C**) A subset of antigens from (**B**) that were previously established to be present on HIV particles are labelled in (**C**), shown here as positive controls for assay specificity.

To use our novel flow virometry screening assay for discovery of new virion-incorporated proteins, we next screened diverse HIV isolates produced in primary PBMC. Here, we limited our focus to screening virion surfaces, instead of cells, since the purpose of using this method was to identify novel virion-incorporated proteins, and PBMC surfaces have already been mapped previously^45^. To begin, we produced four different HIV isolates (BaL, IIIB, SF162 and BG505) in four different PBMC donors (Fig. 2A). We selected these HIV strains for their diversity, since they represent a range of neutralization tiers, encompassing both CXCR4 and CCR5 coreceptor usages, and lab-adapted and transmitted/founder strains. On each of these viral stocks we performed a comprehensive antigen screen with the full panel of 360 antibodies in the LEGENDScreen kit (i.e., 1 screen per isolate; 4 new screens). To compare the level of staining across each virus, we plotted the calibrated mean PE MESF (i.e., PE fluorescence in standardized units) in a heat map in which every row represents a different antigen stain from the screen, and each column represents a different viral isolate (Fig. 2B). A wide range of staining intensities were present on each of the viral isolates, with many visible commonalities and some unique differences seen between the isolates. Since our goal with this manuscript was to identify novel virion-incorporated proteins that had not been described when using traditional techniques, we put less emphasis here on the sources of variation between the different viruses, and more emphasis on the new proteins observed consistently across isolates. We chose to do this since we anticipated proteins that were consistently incorporated were more likely to have a biological impact in vivo. However, it should be noted that some of the variation observed across isolates may be partially attributed to biological differences in protein expression on the PBMC donors, while others may be due to differences inherent to the virus isolate. Indeed, prior work has shown the importance in the host cell profile in determining what is present on the virus, with data demonstrating that genetically identical viruses produced in differential cell types incorporate different host proteins^15,46,47^. Most evidently, HIV_IIIB_ generated in the H9 cell line displayed a staining profile that demonstrated some distinct differences from the same viral isolate cultured in PBMC (comparing column 1 and 2 in Fig. 2B), corroborating that important differences in virion-incorporated protein profiles can occur when identical viruses are passaged in cell lines versus primary cells^48^. Notably, since we stimulate our PBMC with retinoic acid and anti-CD3 (clone OKT3) to make these cells more permissible to infection, some cell surface proteins are likely upregulated on PBMC compared to the unstimulated H9 cells which don’t require any stimuli to become permissive to infection.

To further validate our virus staining results, we displayed a subset of the stains which target proteins known to be incorporated in HIV, providing a broad range of robust positive controls for this assay (Fig. 2C). We have also highlighted a comprehensive list of all positive controls that were previously described in the literature in Supplementary Tables 2 and 3 (with and without isotype subtraction, respectively) for the reader to observe the detection level of these markers with our flow virometry technique. We observed consistent staining for many well-established proteins in the HIV envelope such as HLA-DR, CD43 and multiple tetraspanins (i.e., CD81, CD82, CD63)^25,38^ across all isolates tested. Additionally, host proteins known to have important roles in HIV infection such as ICAM-1, CD44, integrin β7 (β7), CD162, and tetherin (CD317)^6,20–22,49,50^ were also present on multiple HIV isolates. These data suggest that our flow virometry screening assay can reliably detect virion-incorporated host proteins on viruses produced in PBMC, in addition to those produced in cell lines. Notably, since the FV files generated in this assay were calibrated with reference materials and computational software that are well-established in the field^51–53^, our data can reliably be compared to data acquired at different institutions and with different instrumentation.

### Characterizing novel virion-incorporated candidate proteins from the flow virometry screen

To assess the ability of our flow virometry screen to identify novel candidate proteins on the surface of HIV, we generated a list of proteins that stained above isotype on at least three out of four PBMC viruses for further study (Supplementary Table 4). We limited our analyses to proteins that were present on at least three PBMC viruses to reduce the likelihood of including potential false positives from the screen.

**Figure 3:**
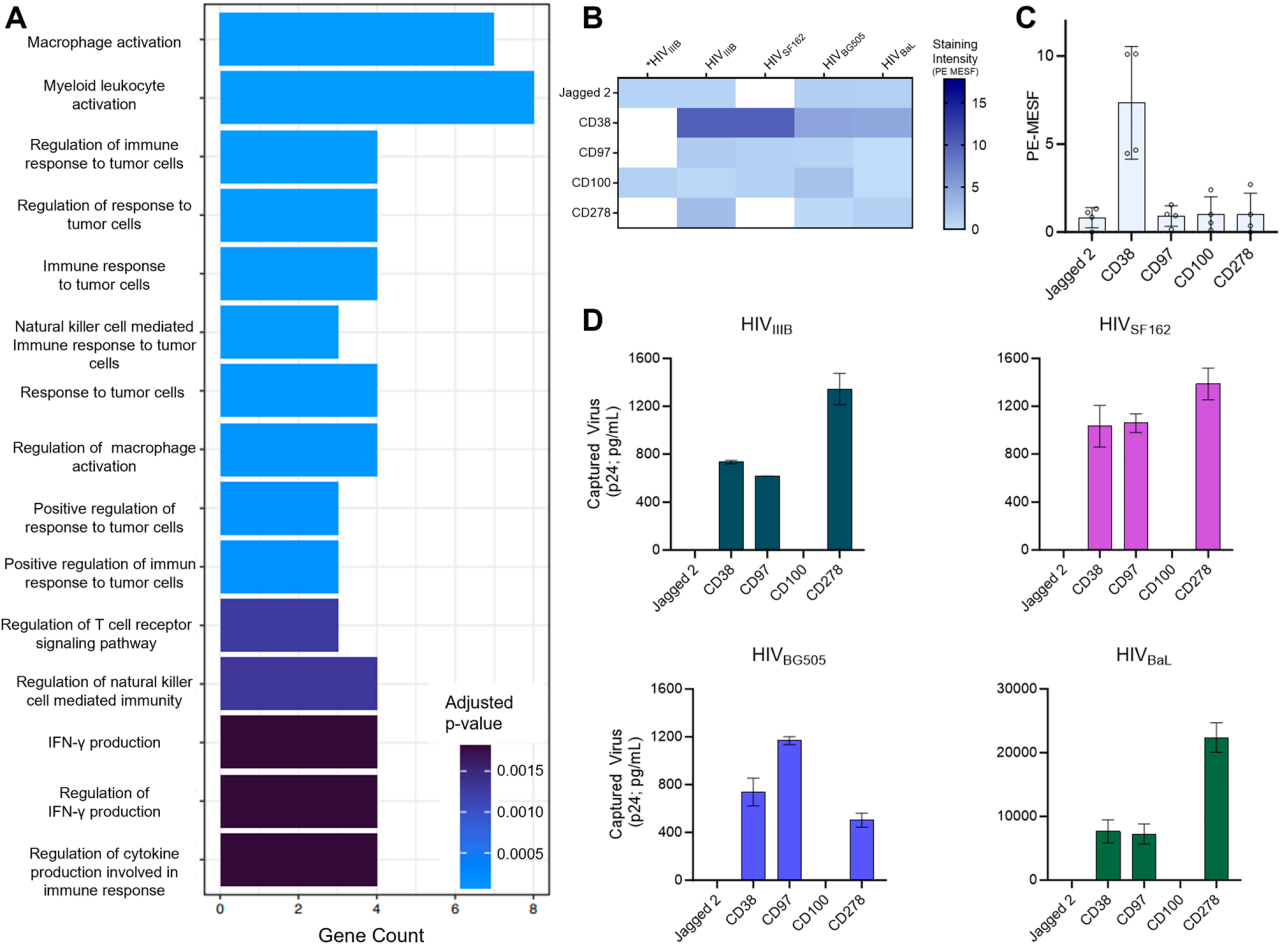
Characterizing novel virion-incorporated candidate proteins identified from the flow virometry screen. (**A**) Purported biological processes of the novel candidates from the LEGENDScreen as described through gene ontology analysis. Novel candidates were defined as proteins that have not been described in the literature on HIV, and those that stained above isotype on at least three out of four PBMC viruses tested. (**B**) A subset of novel candidate proteins identified through the LEGENDScreen. Each row represents a different antibody stain from the LEGENDScreen panel, and each column represents a different viral isolate. *Staining intensities from HIV_IIIB_ produced in the H9 T cell line are shown in column one. Staining intensities for proteins of interest were subtracted from the mean staining values generated from staining with matched isotype controls. Samples with staining below the isotype control are shown in white. (**C**) Graphical representation of the PE MESF values observed in B for the selected novel proteins. Each dot represents a different viral isolate that was tested in the LEGENDScreen. Bars represent the mean PE MESF value ± standard deviation. MESF values with fluorescent intensity below the isotype controls were plotted at zero. (**D**) Virion capture assays were performed to corroborate the presence of the selected novel protein candidates on the viruses. Normalized inputs of the same virus stocks tested in the LEGENDScreen assays (HIV_BaL_, HIV_IIIB_, HIV_BG505_, HIV_SF162_) were incubated with plate-bound mAbs against Jagged 2, CD38, CD97, CD100, and CD278/ICOS. Captured virions were lysed and HIV Gag (p24) was quantified in the lysates (via AlphaLISA), as an indicator of the amount of virus capture. Values are representative of two independent experiments and are reported as the mean ± SD of duplicate AlphaLISA measurements. CD38, CD97, and CD278/ICOS were significantly higher than isotype capture (paired ANOVA; p = 0.001) across all four virus isolates. Virus capture values that were below the isotype control are shown as zero.

The result of this analysis yielded 59 novel candidate proteins, that to our knowledge have not been previously described in the literature to be present on HIV virions. This analysis also identified 36 positive controls that were previously associated with HIV virions through prior mass spectrometry or smaller scale antibody-mediated assays^13,23,24,26^. As a step towards ascertaining the role that these novel candidate proteins may afford HIV when incorporated into virions, we evaluated the novel protein list using bioinformatics. To this end, the gene name associated with each antigen that was determined as a putative novel protein from the screen was run through gene ontology (GO) analysis. By specifically narrowing in on the biological processes that the genes were involved in, we found that many of the novel candidates were associated with cell activation, cytokine production and regulation and responses to tumour cells (Fig. 3A). Given that the viruses were produced in activated PBMC that were responding to viral infection, it is unsurprising that molecules involved with cell activation, signaling and interferon production would be upregulated in these cells. Thus, the proposed biological processes seem highly feasible, though more investigation would be required to ascertain whether these virion-incorporated proteins play any biological role. However, we do caution the interpretation of this GO analysis, as we acknowledge that EVs are contaminating particles within the virus samples tested in flow virometry and the related GO analyses, and therefore, EVs may influence the biological processes reported herein.

From the list of novel candidates identified in the screen, we were particularly interested in highlighting the staining with flow virometry that was observed for CD38, Jagged2, CD100, CD97, and CD278 (inducible T-cell costimulator; ICOS) on virus particles (Fig. 3B and C). We narrowed our focus to these proteins due to their reproducibility across multiple isolates (present on at least 3 of 4 primary isolates tested), moderate/high levels of staining, and the potential impact these proteins may have on HIV infection due to their inherent biological properties. To corroborate the presence of these newly discovered proteins on virus particles, we employed a plate-based virus capture assay to precipitate virions that incorporate those proteins on their surface. Importantly, the virus capture assay affords the advantage of selectively detecting viruses over contaminating EVs because the readout for this assay is quantification of the viral capsid protein, HIV Gag p24, in capture assay lysates. As discussed earlier, we acknowledge that EVs will contribute to some of the observed virus staining with flow virometry techniques, due to the highly similar antigen profiles and biogenesis pathways both particle types share. However, since the viral capsid protein (HIV Gag p24) has been shown to be present at very low levels in non-virus vesicles^54,55^, it can help in distinguishing antibody-mediated capture of virus particles versus EVs.

Across all isolates tested, CD38, CD97, and CD278/ICOS were present at significantly higher levels than background capture, assessed by capture with an isotype-matched control antibody (Fig. 3D). These data confirmed that CD38, CD97, and CD278/ICOS are indeed novel antigens present on the surface of virus particles produced in PBMC. Importantly, we also observed that antibodies against Jagged2 and CD100 did not capture any appreciable levels of virus, indicating that the staining observed with flow virometry screening techniques for Jagged2 and CD100 may have represented predominant staining on EVs, rather than virus particles specifically. These data emphasize the importance of validating flow virometry analyses with orthogonal techniques to help distinguish virus particles from extracellular vesicles. Taken together, these data also highlight the utility of our novel flow virometry assay for identifying new candidate proteins on the HIV surface, in a high throughput manner.

For the purpose of this manuscript, we were interested in focusing on the discovery of virion-incorporated CD38, due to its exceptionally high abundance on virions observed in the flow virometry and capture assays, and for the known role CD38 plays as a prognostic marker in HIV infection^31^). Indeed, CD38 is abundant on activated lymphocytes, which has previously been linked to increased risk of progression to AIDS in adults^56^. Furthermore, the molecule’s ability to serve as an ectoenzyme, adhesion receptor and signalling molecule^30^ could suggest that its incorporation in the HIV envelope may afford virions a biological advantage. Due to this, we decided to narrow our further analyses to this protein.

### Bead-based virion capture assays corroborate the presence of CD38 on viruses

**Figure 4:**
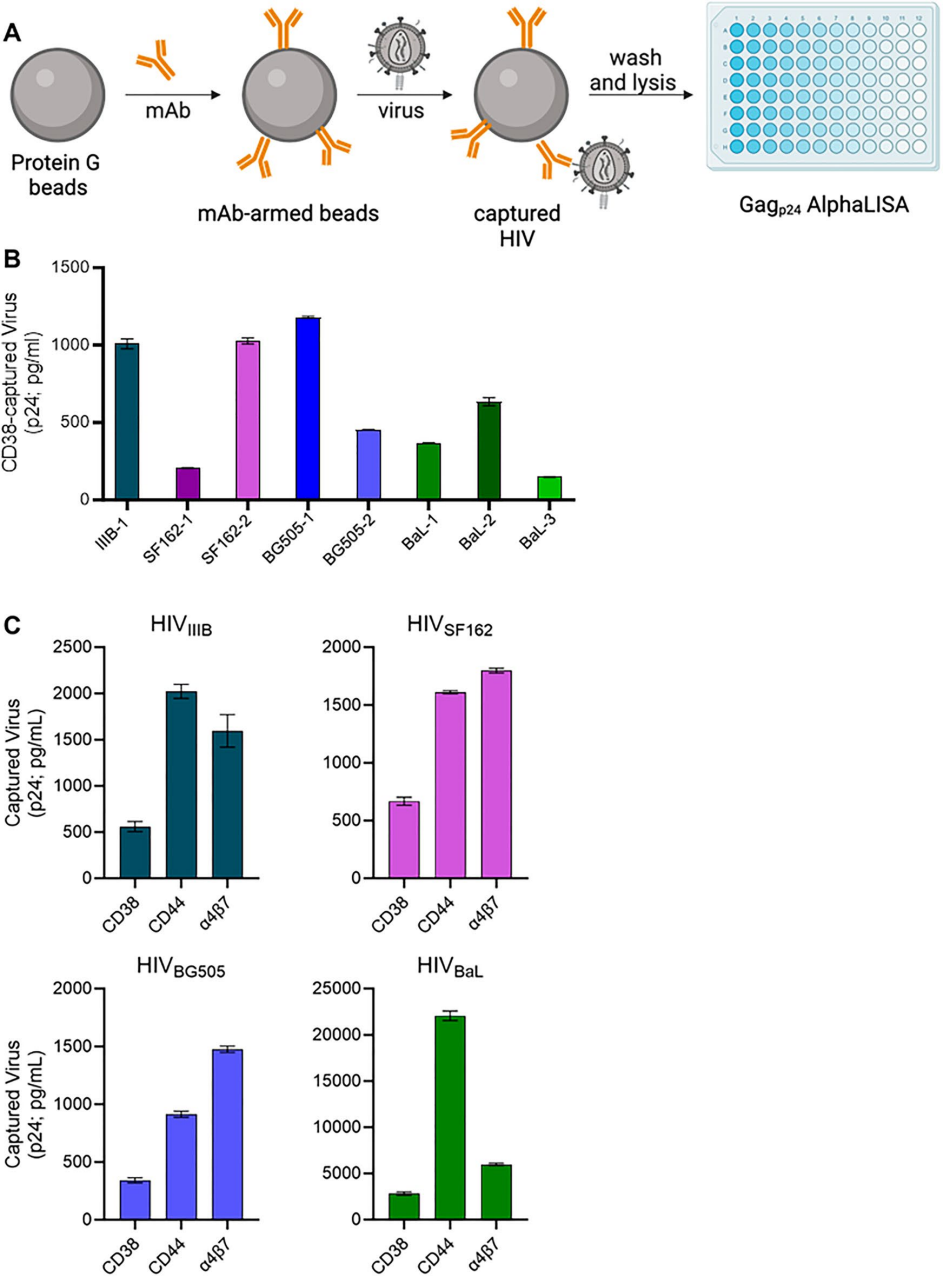
Bead-based capture of HIV virus stocks using an anti-CD38 antibody. (**A**) A schematic representation of the bead-based virion capture assay. Protein G coated magnetic beads are armed with the antibody (mAb) against the protein to be detected on the viral surface. The virus is incubated with the antibody-armed beads for 2 h at room temperature to allow virus capture, followed by washing and lysis of bead-bound (i.e., captured) virus. The amount of virus captured via each mAb is quantified by Gag p24 AlphaLISA performed on viral lysates. (**B**) Virion capture assays were performed on equal volumes of eight independent virus stocks of HIV isolates (BaL, IIIB, BG505, SF162) grown in different PBMC donors. The numbering of isolates indicates a distinct viral stock produced in a different PBMC donor for the same viral isolate (i.e., BaL-1, BaL-2, and BaL-3 are the same BaL isolate grown in 3 different PBMC donors). Bead-associated virus was lysed and HIV-1 p24 Gag was quantified using p24 AlphaLISA as an indicator of the amount of virus capture. Background levels of capture achieved with an isotype-matched control antibody were subtracted from all data shown. Levels of CD38 capture displayed are significantly higher than isotype capture (paired t-test; p = 0.003). (**C**) Virion capture assays were performed with immunomagnetic beads armed with antibodies against CD38, CD44 or integrin β7 with each virus isolate (BaL, IIIB, BG505, SF162). In parallel, we assessed the levels of background capture as measured with an isotype control, and these control values were subtracted from the capture data before graphing. Levels of CD38 capture displayed are significantly higher than isotype capture (paired ANOVA; p = 0.004). The mean ± SD are generated from independent virus stocks tested in duplicate.

To corroborate the presence of CD38 on virions with a complementary technique, we performed bead-based virion capture assays (Fig. 4A). This method captures native viruses in suspension from culture supernatants, using Protein G beads armed with mAbs against human proteins, as previously performed^6,34,57,58^. After washing away unbound virus, the bead-captured virions are lysed for a readout of Gag p24, thereby enabling this technique to confirm the detection of CD38 on HIV virions (with their associated Gag p24) and not contaminating EVs. As discussed earlier, capture assays afford some distinct advantages in specifically analysing virus particles within a sample, despite contaminating EVs being present in the viral stocks used.

To begin, we probed for the presence of CD38 on equal volumes of eight different undiluted virus stocks (80–120 ng/mL of p24), representing 4 different viral isolates produced in PBMC, as labelled. The numbering of isolates in Fig. 4B indicates a viral stock produced in a different PBMC donor for the same viral isolate (i.e., BaL-1, BaL-2, and BaL-3 represent the same BaL isolate grown in 3 different PBMC donors). We performed bead-based capture assays with immunomagnetic beads armed with anti-CD38 antibody to capture virions with incorporated CD38 (Fig. 4B). Across all isolates tested, CD38 was present at significantly higher levels than background capture, which was assessed by an isotype control antibody. The level of the isotype control is subtracted before graphing the data to allow for ease of comparison across the isolates which have different viral titres. To compare the relative abundance of CD38 to other host proteins known to be present in the HIV envelope, we performed capture assays on the same four viruses that were tested in the LEGENDScreen (BaL, IIIB, BG505, SF162), using anti-CD44, anti-integrin β7 and anti-CD38 in parallel (Fig. 4C). CD38 was found to be present on all four isolates, but at consistently lower levels than both of the other host proteins tested. This finding could suggest that CD38 is less abundant than the other proteins, or that it is less accessible to antibodies in this type of capture assay. Despite these considerations, these data provide confirmation that CD38 is new molecule of interest present on HIV and highlights the utility of our flow virometry assay for identifying novel candidate proteins on the HIV surface that can be validated through orthogonal techniques.

### Quantitative assessment of CD38 levels on HIV using flow virometry

After validating the presence of CD38 on virions using bead-based capture assays, we sought to use FV techniques to quantify the number of CD38 molecules on virions using FV. Although the flow virometry screening technique accurately identified many well-established proteins and novel candidate proteins in the HIV envelope, because of the nature of the large screening assay, we were unable to titrate the antibodies against each antigen. While we diluted the antibodies provided in the LEGENDScreen kit to a range that was suitable for staining small particles, staining with optimal antibody concentrations is required to ensure accurate protein quantitation on virions. Furthermore, because our flow virometry protocols do not involve a wash step, optimizing the fluorescent signal while minimizing excess antibody is paramount. For this purpose, we performed a 7-point antibody titration to ascertain a concentration that provides robust staining with low levels of background (Supplementary Fig. 1). Next, we performed serial dilutions of CD38 stained virus (Supplementary Fig. 2) to ensure the cytometer was accurately analyzing single particles, with the acknowledgement that individual particles analysed could represent viruses and/or extracellular vesicles. From plotting PE molecules of equivalent soluble fluorophore (MESF) versus particle count from a stained population of vesicles, we observed that the level of PE fluorescence stayed consistent across multiple dilutions, while the particle number decreased in a two-fold manner (Supplementary Fig. 2B). This trend is consistent with the analysis of single particles since it demonstrates that the amount of fluorescence is being contributed by single particles, not aggregates of particles, which would not dilute in a linear fashion^51^.

**Figure 5:**
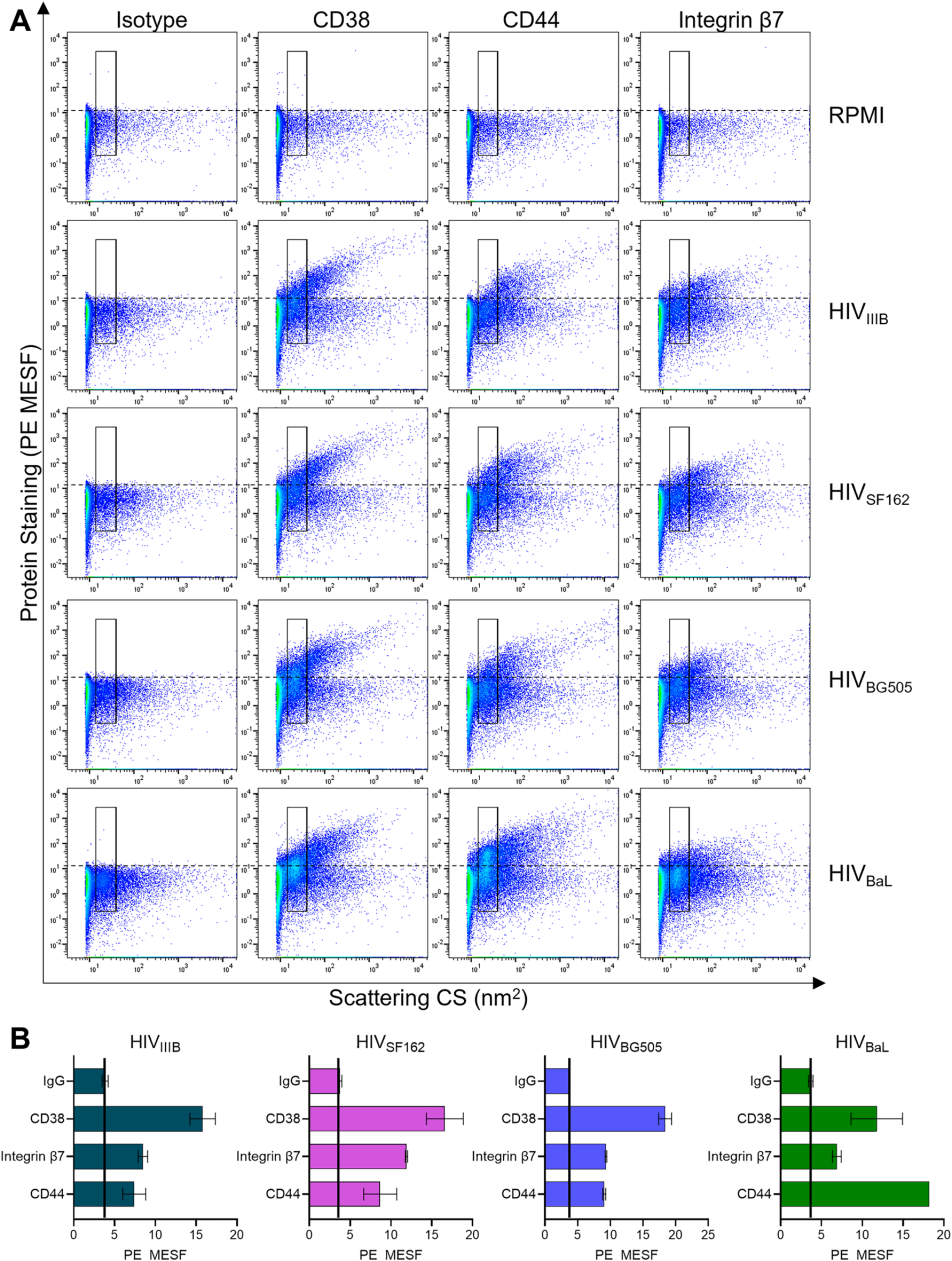
Quantitative flow virometry staining of CD38 on the surface of HIV. (**A**) Four different HIV isolates (IIIB, BG505, SF162, BaL) produced in PBMC were stained with PE-conjugated antibodies against CD38, CD44 and integrin β7. A stained cell culture medium control (RPMI) is shown to assess the contribution of antibodies alone (in absence of virus) to background fluorescence. Gates demarcate where the majority of viruses are anticipated to fall based on light scattering. Dotted lines indicate the level of background PE fluorescence set based on the isotype control. (**B**) Quantitative representation of mean fluorescent data (PE MESF) ± SD generated through the gates in two experimental replicates from flow virometry data as in (**A**). Solid black lines indicate levels of background capture as measured with an isotype control. Data shown are representative of five replicates for each virus stock.

Next, we stained for CD38, CD44 and integrin β7 on the surface of PBMC viruses using the titrated antibody conditions (Fig. 5). Based on our quantitative staining protocols^34^, the calibrated PE MESF values obtained from staining can provide estimates regarding the number of antibodies bound to a particle. This comes with the assumptions that no steric hindrance obstructs antigen binding, and the validated assumption that each antibody has only one conjugated molecule of PE due to the large size of this fluorophore^59–61^. Although this quantitation can vary based on instrument sensitivity and gating strategy, we can estimate that staining MESF values are as close as ~ two-fold from the absolute number of proteins on the virion surface based on the bivalent nature of antibodies^59–61^.

For the three host proteins tested, we saw that robust levels of staining were present on all of the virus samples, but not in the cell culture media controls (RPMI). This demonstrated that the labelling seen was specific to our samples and not non-specific fluorescence from antibody aggregates. Interestingly, different staining distributions were observed relative to the type of host protein stained, with the lowest levels of staining seen for integrin β7. Through the use of a gate specific to our virus population, as determined by virus scatter^34^, we generated mean fluorescence values for comparisons of incorporated proteins on the different virus stocks (Fig. 5B). From these data CD38 was shown to be the most abundant host protein on all of the isolates tested, with MESF values averaging between 12 ± 3 MESF and 18 ± 1 MESF, while noting that this estimate of protein count may be off by a factor of two due to the bivalent nature of antibodies. Interestingly, CD44 staining ranged between 7 ± 1 MESF and 9 ± 1 MESF for all virus isolates except for HIV_BaL_, where it was considerably higher, reaching 18 ± 1 MESF. This was in line with virion capture results where CD44 was captured to a high degree on HIV_BaL_ (Fig. 4C). Staining of integrin β7 with FV methods was more modest than the levels of relative capture observed in Fig. 4B. In line with our results, others have shown that the same monoclonal antibody (mAb) used in parallel across different assays can yield diverse results related to analytical interference, whereby changes in antibody specificity and accuracy are observed due to the different experimental conditions^62^. Studying how mAbs perform in parallel across FV and virus capture assays is ongoing work in our lab (manuscript in preparation).

Nevertheless, FV is inherently more susceptible to the contributions of extracellular vesicles, especially when compared to our capture assay which uses viral Gag p24 as a readout. To address this, we assessed the contribution of EVs to our flow virometry staining (Supplementary Fig. 3). To this end, we stained cell culture supernatants from uninfected PBMC that were cultured the same way as our virus producer cells and compared them to stained viruses. As expected, we saw that EV staining was present for the cellular antigens in the cell culture supernatants in the absence of virus, indicating that EVs can also contain many cellular antigens incorporated into virus particles. However, while EVs are known to share many of the same markers as viruses^63^, differences can be observed in the scatter profiles of our virus preparations compared to vesicles alone (Supplementary Fig. 3), as also seen previously in our work^22^. Additionally, although the samples were acquired for an equal amount of time, virus preparations contained ten-fold more particles than seen in the EV control condition (Supplementary Fig. 3), indicating that the notable differences in particle concentration can be detected by the cytometer for these samples. Therefore, although vesicle staining may contribute to some of the fluorescence we attributed to CD38 staining on viruses, the use of appropriate flow virometry controls, in tandem with complementary techniques like virion capture assays, corroborates that our flow virometry screening technique can reliably identify novel candidate proteins on the surface of enveloped virions.

## Discussion

Host proteins on the surface of viruses can play important roles in viral infection, and the first step towards characterizing these roles is to find effective ways to identify the proteins. Herein, we developed a novel flow virometry screening assay based on a commercially available cellular staining kit to innovate the discovery of human proteins on viruses. Our goal was to identify novel proteins in the HIV envelope using an alternative experimental approach that allows for a high throughput, cost effective screen of cellular antigens on the HIV surface. We found that our FV screening assay allowed for the identification of many novel proteins in the HIV envelope, in addition to identifying many well validated controls. Importantly, our flow virometry methods herein are robust and reproducible, given that we employed a suite of well-established calibration tools and guidelines (MIFlowCyt-EV framework^51^) for nanoparticle analyses. The guidelines which we have followed ensure that this work can easily be reproduced by other laboratories to spur scientific progress in both the virology and extracellular vesicle fields.

While host proteins on HIV have been studied for decades, host proteins on other classes of viruses remain largely understudied. The protocols shown here can feasibly be applied to other viruses which have not been studied extensively, with a high potential for new discoveries. For such viruses, performing complementary screens of the producer cells may also be highly valuable to gain insight on whether certain proteins are being selectively enriched or depleted from the viral envelope. This was evident here from CD45 staining which is highly abundant on H9 cells but was relatively sparse on virions. Importantly, although our analyses focused on viruses, this technology could easily be applied to extracellular vesicles as well, due to the similarities they share in size and antigen profile with viruses.

Through our flow virometry screening assay we were able to identify 59 novel candidate proteins incorporated on HIV virions. However, it should be noted that the LEGENDScreen kit used herein is a targeted antibody panel, that is restricted to analyzing only 400 leukocyte antigens, and many proteins that exist on viral producer cells were not tested in this limited study. Additionally, while the specific antibody panel used here allowed for the ease of staining and a broad selection of antibodies, identical work could be performed with individual antibodies or similar proprietary kits. Notably, the reported number of candidates is likely to represent an underestimate for several reasons: (1) proteins present at low abundance on virions may not be detected on our cytometer due to its inherent limit of sensitivity (~ 10 MESF); (2) novel candidate proteins that were present on only two PBMC viruses were not included in our gene ontology analysis to increase stringency; (3) proteins that stained at levels which were similar or slightly below the isotype controls were not considered. Nevertheless, the power that FV shows to identify proteins on the viral surface is clearly demonstrated herein through the number of positive controls identified in the assay.

Our results showed that diverse virus isolates produced in different PBMC donors shared a high number of common virion-incorporated proteins. Furthermore, reproducibility across the same virus isolate (H9 HIV_IIIB_) from two different screens (biological replicates) contained 70% of the same top staining targets, despite the two assays being performed with different acquisition parameters (threshold and sample dilution). This is notable since one of the caveats of large-scale protein screening techniques is a potential for lack of reproducibility. Indeed, while mass spectrometry has been used to identify many proteins in the HIV envelope^13,64^, a concerted effort in the field is required to address technical issues which can hinder experimental reproducibility^65–68^. The flow virometry techniques herein offer distinct advantages for reproducibility by pairing FV with calibration, using commercially available reference materials and publicly available computational software. Indeed, the field of small particle flow cytometry is well equipped with standards for reproducibility and have detailed guidelines for standardized reporting and operating practices^51,69,70^. With these tools in hand, our calibrated data can be quantitatively compared across different samples, instrumentation, and institutions.

While our novel flow virometry assay provides many advantages for robust and reliable data reproducibility, our results herein do show some biological variation across the diverse isolates tested. Since the four virus isolates used in this study were propagated in primary cells from four different human blood donors, the levels of variation observed here could be due to differential cell surface expression and/or strain specific differences in virion incorporation, both of which were not explored herein. Notably, the subtraction of background fluorescence as detected by isotype control antibodies could also contribute to an underestimated reading of host protein abundance on virions, leading us to report the absence of proteins on virions due to their low levels of incorporation. However, since our goal with this manuscript was to discover new virion-incorporated proteins of interest using a novel flow virometry screening assay, we focussed on reporting the proteins that were consistently incorporated across the majority of isolates tested, rather than identifying the sources of variation.

In this manuscript we evaluated viruses from just four different primary donors, but posit that by generating a database of protein profiles from viruses from a wider range of donors, the implications of the work could be far reaching. For example, if enough information is known about how the virion surface can correlate to the state of its producer cell, then diagnostics and monitoring of disease progression could be further innovated with this knowledge. Moreover, being able to determine the phenotype of a virus’ producer cell from the viral protein profile could be particularly useful for identifying and monitoring the HIV reservoir. While these ideas are not within our current scope, progress is being made in this area in the extracellular vesicle field. Indeed, the use of biomarkers on biological nanoparticles is currently being studied for personalized medicine and improved treatment and monitoring of disease, particularly in cancer research^71–76^.

Although we were able to confirm the presence of CD38, integrin β7 and CD44 using both FV and virion capture, there were differences in the relative amounts of host protein detection across these techniques. This could be a combined effect of differential contributions of EVs in the assays, and/or different antigen accessibility when viruses are bound to beads or are in free suspension. Additionally, after subtracting levels of background fluorescence with an isotype control antibody in our flow virometry screening assay, we did not detect CD38 on the viruses that were propagated in H9 cells, despite CD38 being present on all viruses propagated in PBMC. However, when we performed more targeted experiments with optimized antibody concentrations for FV staining and virion capture assays, we did observe CD38 on virus particles produced in H9 cells, albeit at very low levels that are close to threshold for detection. Investigating the differences in methodology to evaluate host proteins on virion surfaces is an active area of investigation by our group.

Finally, while we identified many novel candidate proteins through this screening assay, we did not seek to extensively characterize any of them herein. Although we did validate that CD38, CD97, and CD278/ICOS were present on HIV using a complementary technique, the purpose of this manuscript was to highlight the ongoing potential for discovering novel virion-incorporated proteins with flow virometry, rather than the characterization of any individual protein. Nonetheless, it is tempting to speculate what additional biological properties the newly discovered proteins (CD38, CD97, and CD278/ICOS) might afford HIV particles when incorporated on the virus surface. For example, since CD38 is known to bind both CD31 and CD16 in a cellular context, it is possible that virion-incorporated CD38 may offer enhanced adhesion properties to virions in vivo. Similarly, because CD38 is a known prognostic marker of HIV infection on cells^31^, it would be interesting to see if it could be a biomarker of use when present on virions. Similar to our prior work on virion-incorporated integrin α4β7^6^, it is possible that CD97 (adhesion G protein-coupled receptor E5) on the surface of virus particles could influence virus homing, particularly to endothelial cells expressing its cognate ligand, CD55^77^. Previous studies have shown that CD97-CD55 interactions between monocytes and T cells can elicit robust costimulatory signals^78^, and therefore, viruses with CD97 on their surface may also benefit in enhanced attachment at immunological synapses. Furthermore, our discovery of virion-incorporated CD278/ICOS, the Inducible T-cell costimulator, may also promote virus retention at immunological synapses, given the many well-established co-stimulatory roles of ICOS on T cells, and via interactions of virion-bound ICOS with its cognate ligand, ICOS ligand (ICOSL), on antigen presenting cells (APCs)^79,80^. Beyond just influencing viral attachment, virion-incorporated CD278 may also block T cell activation by binding to ICOSL on APCs and preventing the delivery of co-stimulatory signals, similar to the ability of incorporated PD-L1 to reduce T cell proliferation and IL-21 production^12^. These intriguing speculations and other biological questions related to the novel candidate proteins identified from the screen remain the scope of future work in our group.

## Methods

### Cell culture

The H9 T cell line (isolated from a 53-year-old male) and PBMC of unknown biological sex were used to produce virus through infection were maintained in RPMI-1640 (Wisent, Cat#350-000-CL) with 10% heat inactivated fetal bovine serum (Wisent, Cat#098150), 100 U/mL penicillin, and 100 µg/mL streptomycin (Life Technologies, Cat#15140122). All cells were grown in a 5% CO_2_ humidified incubator at 37 °C. Primary cells were collected through the NIH Department of Transfusion Medicine protocol that was approved by the Institutional Review Board of the National Institute of Allergy and Infectious Diseases, National Institutes of Health. All methods were performed in accordance with the relevant guidelines and regulations. Informed consent was obtained from all study participants.

### Virus production

For infection, H9 CD4^+^ T cells were pelleted and resuspended in 1 mL of HIV^IIIB^ virus stock (150 ng of total p24) for 4 h at 37 °C. Afterwards, fresh media was added to the cells which were grown in a T75 flask until the time of harvest. For primary cells, PBMC were activated with anti-CD3 (clone OKT3), IL-2 (20 IU/mL), and RA (10 nM) for 3 days before infection. Activated PBMC were cultured in 6 well plates, and for each well 5 ng of p24 for each virus stock (HIV_BaL_, HIV_IIIB_, HIV_SF162_ or HIV_BG505_) was used. During infection, every 3 days fresh media with IL-2 and RA as above was used to replace half of the media in the wells. Cell culture supernatants containing virus were harvested 7–12 days later, based on dates of peak viral titre. Viral titres were quantified by p24 measurement with high sensitivity AlphaLISA (Perkin Elmer). Virus containing culture supernatants were centrifuged for 5 min at 300×*g* to remove cellular debris before being stored at − 80 °C until use, without any filtration of the supernatants. All viral stocks used for virus production in this study were previously adapted to the cell type used herein (H9 T cells or PBMC), to ensure productive infection and virus growth for the purpose of this study. Thus, virus stocks that were previously passaged in PBMC were used to generate new PBMC-derived viruses assayed in this study, and an HIV_IIIB_ stock that had been previously passaged through H9 cells was used to generate new H9-derived HIV_IIIB_ virus assayed herein.

### Flow virometry

Flow virometry was performed using a Beckman Coulter CytoFLEX S with standard optical configuration. The PE Gain and threshold optimization for detection of virus and calibration beads was performed as described previously^53^. Viruses run in the LEGENDScreen were acquired for 30 s while viruses used for quantitative flow staining were acquired for 2 min at a sample flow rate of 10 μL/min. For labelling, cell-free supernatants containing virus were stained at to 10^8^ particles/mL overnight at 4 °C with PE-conjugated antibodies against CD44(BD Cat# 550989), CD38 (BioLegend Cat# 303506) or integrin β7 (BioLegend Cat# 321204). After staining viruses were diluted two-fold with 4% PFA (2% final) for 20 min for fixation. After fixation, all samples were further diluted with PBS to reduce coincidence before FV analysis. BD Quantibrite PE beads (CA, USA; Cat#340495, lot 91367) and NIST-traceable size standards (Thermo Fisher Scientific) were used for fluorescence and light scattering calibration respectively (see Supplementary Figures). Calibration was performed using FCM_PASS_ software (https://nano.ccr.cancer.gov/fcmpass) as previously described^52,53^. Detailed information on the fluorescent and light scatter calibration and the MIFlowCyt-EV checklist^51^ can be found in the FCM_PASS_ output report in the Supplementary Figures composite file**.** All data were analyzed using FlowJo software version 10.7.1. (CA, USA). PE MESF statistics were generated from gates set on the virus population using FlowJo.

### LEGENDScreen

H9 T cells were stained using the BioLegend LEGENDScreen kit (Cat# 700007) following the manufacturer’s instructions. Briefly 500,000 cells were added to each well of a 96 well plate in a volume of 75 μL. Next, the LEGENDScreen antibodies were added to each well following the plate layout provided by the company. Cells were stained for 30 min at 4 °C in the dark before being washed with PBS and fixed with fixation buffer. Fixed cells were transferred to polystyrene tubes and were acquired on a CytoFLEX S cytometer. For virus staining the following modifications to the manufacturer’s protocol were made. After reconstituting the LEGENDScreen’s lyophilized antibodies with sterile water, the antibodies were further diluted twofold in water. Next, antibodies were further diluted 25-fold in new U-bottom 96 well plates in PBS. Viruses were added to the new plates at a 1:1 ratio with antibody and left to incubate overnight at 4 °C in the dark. Finally, viruses were fixed with 4% PFA before being diluted in 4 mL glass tubes (Fisher Scientific Cat#14-961-25) with PBS for acquisition. Heat maps were generated with Prism 9.5.0 (GraphPad, San Diego, CA, USA) using the mean PE MESF values of the stained antigen after subtracting the MESF value of the isotype control.

### Gene ontology analysis

A list of genes encoding the proteins that were identified as being present on at least three out of four PBMC viruses from the LEGENDScreen were used as input for Gene Ontology (GO) analysis. The analysis was conducted with a threshold of adjusted p-value < 0.05 (Benjamini-Hochberg), using the *enrichGO* function from the clusterProfiler package^81^ with the org.Hs.eg.db database^82^. Enrichment results denoting the associated biological processes were sorted by the adjusted p-value, and the top fifteen statistically enriched processes were visualized using enrichplot and ggplot2^83^.

### Virion capture assay

A plate-based virion-capture was performed by coating flat-bottom 96-well microtiter plates with 5 µg of anti-CD38 (clone HB7), anti-CD97 (clone VIM3b), anti-CD100 (clone A8), anti-Jagged 2 (clone MHJ2-523) and anti-CD278 (clone C398.4A) antibodies overnight at 4 °C. The plates were then washed with PBS and blocked with 10% BSA. After blocking, plates were washed with PBS and incubated with 50 µl of normalized virus inputs (100 ng/mL of p24), for overnight incubation at 4 °C to allow virus capture. Plates were then washed 3 times with PBS to remove unbound virus. The plate-associated virus was lysed with 50 µL of 0.5% Triton X-100, followed by p24 quantification in the viral lysates with AlphaLISA. Data analysis was performed using Prism 9.5.0. The background level of virion capture for each virus type was assessed by virion capture with an isotype-matched control antibody (BD Cat# 557273 and BioLegend Cat# 400901). Levels of background capture using the isotype antibody were subtracted from all datasets shown, as indicated.

Immunomagnetic bead-based virion capture was performed as previously described^6,57^. In brief, for each reaction, 25 μL of protein G Dynabeads (Life Technologies; Cat#10004D) were armed with 0.5 μg of anti-CD38 (clone HB7), anti-CD44 (clone 515), or anti-a4b7 (clone ACT-1) antibodies for 30 min at room temperature, and then washed with PBS containing 10% FBS to remove unbound antibodies. Normalized inputs of virus were tested, with all viral stocks starting at 35 ng/mL of p24, except for HIV_BaL_ which was used at its undiluted titre (135 ng/mL). For all tests, equal volumes (150 μL) of each virus were incubated with antibody-armed beads for 2 h at room temperature to allow virus capture. Beads were then washed twice with 10% FBS–PBS, and one final wash with 0.02% FBS–PBS to remove unbound virus. The bead-associated virus was lysed with 0.5% Triton X-100 for p24 quantification by AlphaLISA. Data analysis was performed using Prism 9.5.0. The background level of virion capture for each virus type was assessed by virion capture with an isotype control antibody (BD Cat# 557273). Levels of background capture using the isotype antibody were subtracted from all datasets shown, as indicated.

### p24 AlphaLISA

The quantification of HIV-1 p24 capsid protein was performed in captured virus lysates with the AlphaLISA p24 detection kit following the manufacturer’s (PerkinElmer) instructions. Endpoint Alpha light emission readings were performed on a Synergy NEO 2 multimode plate reader equipped with the Alpha filter cube (BioTek, VT, USA) using Gen 5 software (v. 3.08).

### Statistical analysis

Statistical analysis was performed using GraphPad Prism. Analysis of Fig. 4A was conducted by a two-tailed, paired Student’s t-test with P = 0.003. Analysis for 4B was conducted by paired ANOVA with p = 0.004. Error bars for all data sets represent mean ± standard deviation with the number and nature of replicates indicated in the figure legends.

### Supplementary Information

Supplementary Figures.

Supplementary Tables.

## Data Availability

All data is available from the corresponding authors upon request.
