# Supplementary material for: Identification of CD38, CD97, and CD278 on the HIV surface using a novel flow virometry screening assay
Source: Sci Rep. 2023 Dec 27;13:23025. doi: 10.1038/s41598-023-50365-0 (PMC10754950; doi:10.1038/s41598-023-50365-0)
Supplement: Supplementary file 1 — Supplementary Figures. [file 41598_2023_50365_MOESM1_ESM.pdf]

Fig. S1. Antibody titration of flow virometry antibodies. (pg 1/2)

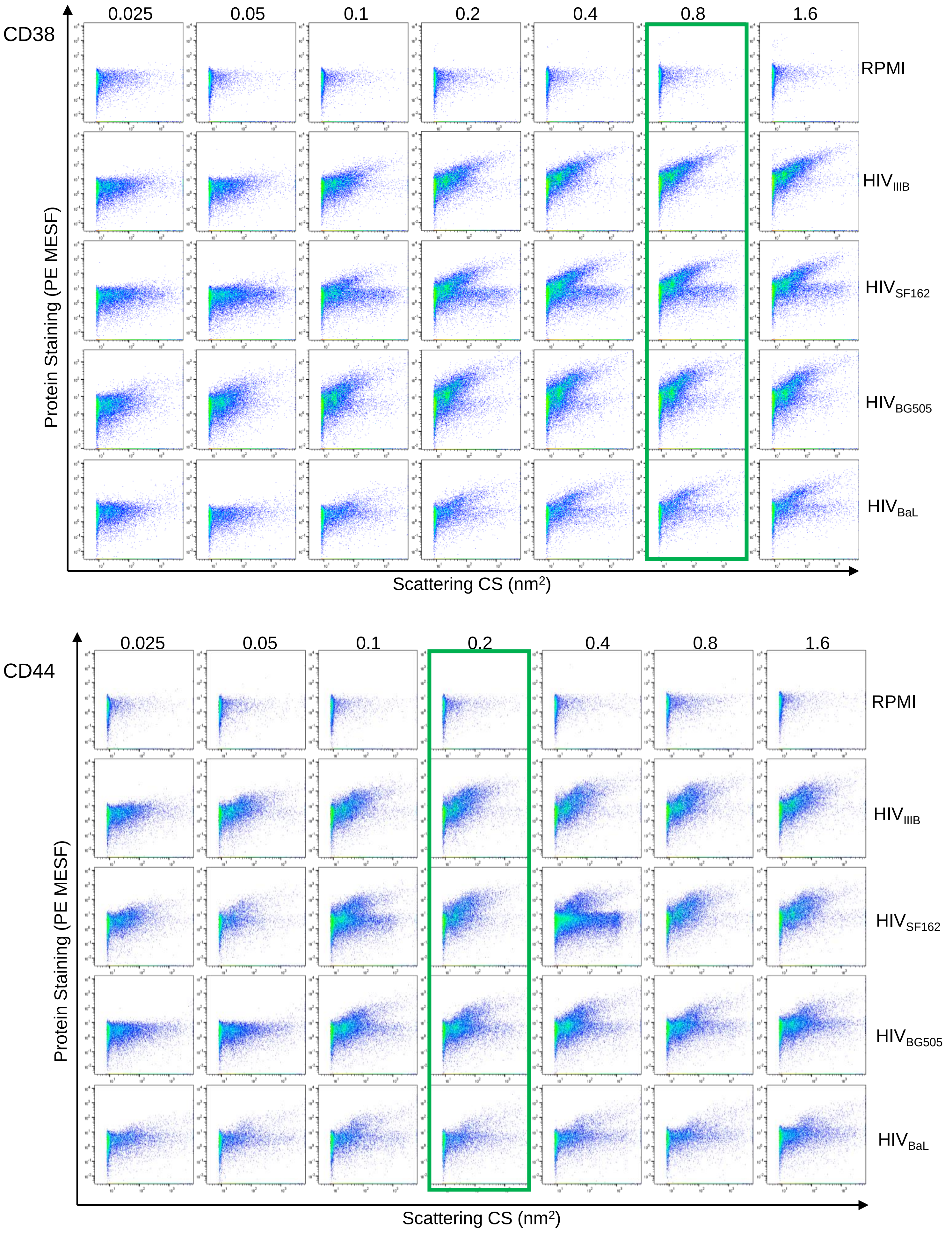

Fig. S1. Antibody titration of flow virometry antibodies. (pg 2/2)

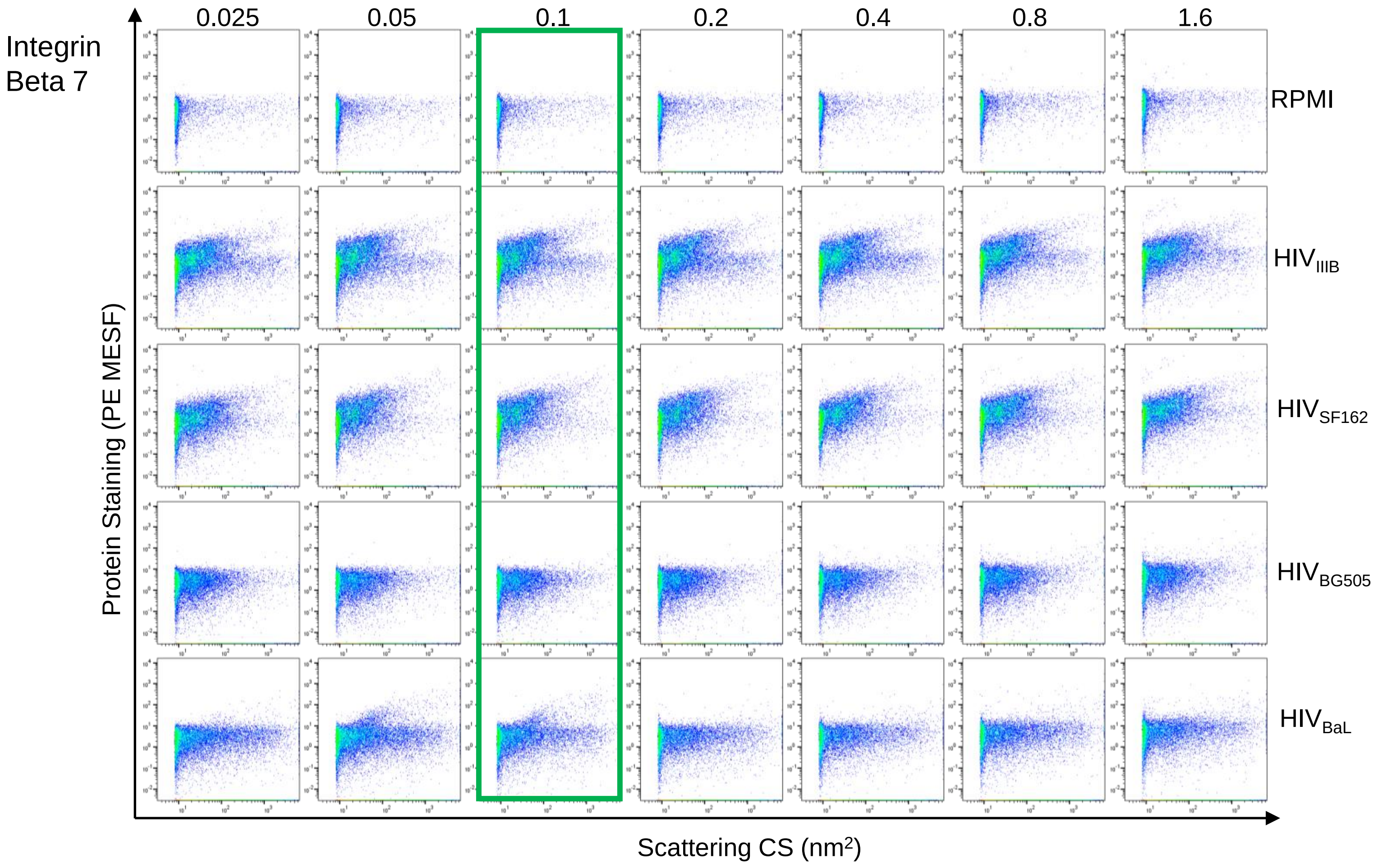

Fig. S1. Antibody titration of flow virometry antibodies (related to Figure 5). Pseudocolour dot plots of virus samples stained with different antibody concentrations (0.025-1.6 µg/mL) of anti-CD38 PE, anti-CD44 PE and anti-integrin β7 PE. The antibody concentration that was selected for use in further experiments is shown in green, and was based on optimal staining intensity and minimal background fluorescence across multiple isolates tested. A stained cell culture medium control is shown to assess the contribution of antibodies alone (in absence of virus) to background fluorescence.

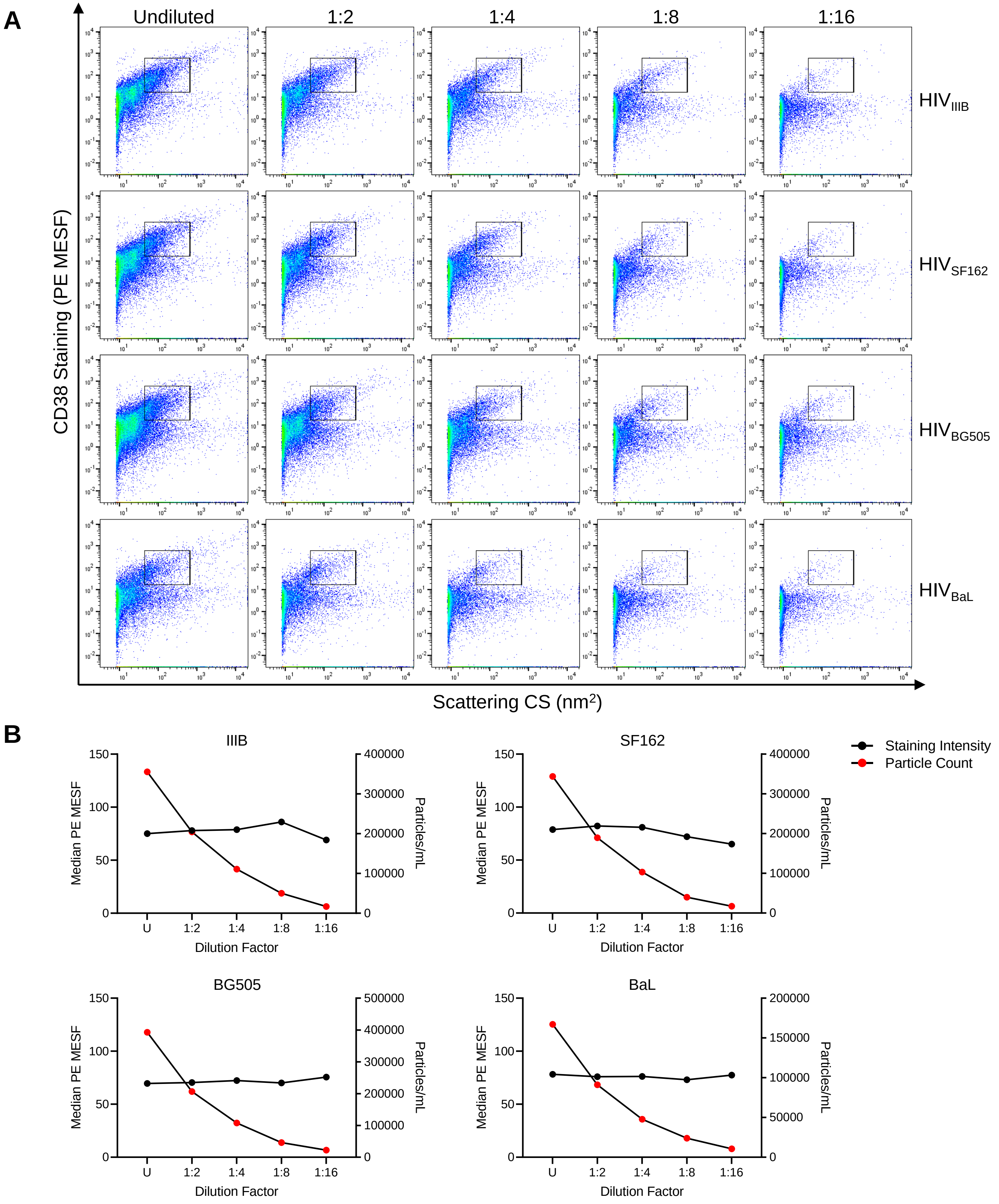

Fig. S2. Serial dilutions of CD38 PE-stained virus samples (related to Figure 5). (A) Pseudocolour dot plots of virus samples (BaL, BG505, IIIB, SF162) stained with an anti-CD38 PE antibody. Stained samples were serially diluted two-fold to a factor of 1:16 to assess single particle detection. Gates were set on a stained population of vesicles that are well above background to demonstrate that the intensity of staining stays constant over time. (B) Quantitative median PE MESF values and particle counts from (A) for each virus sample.

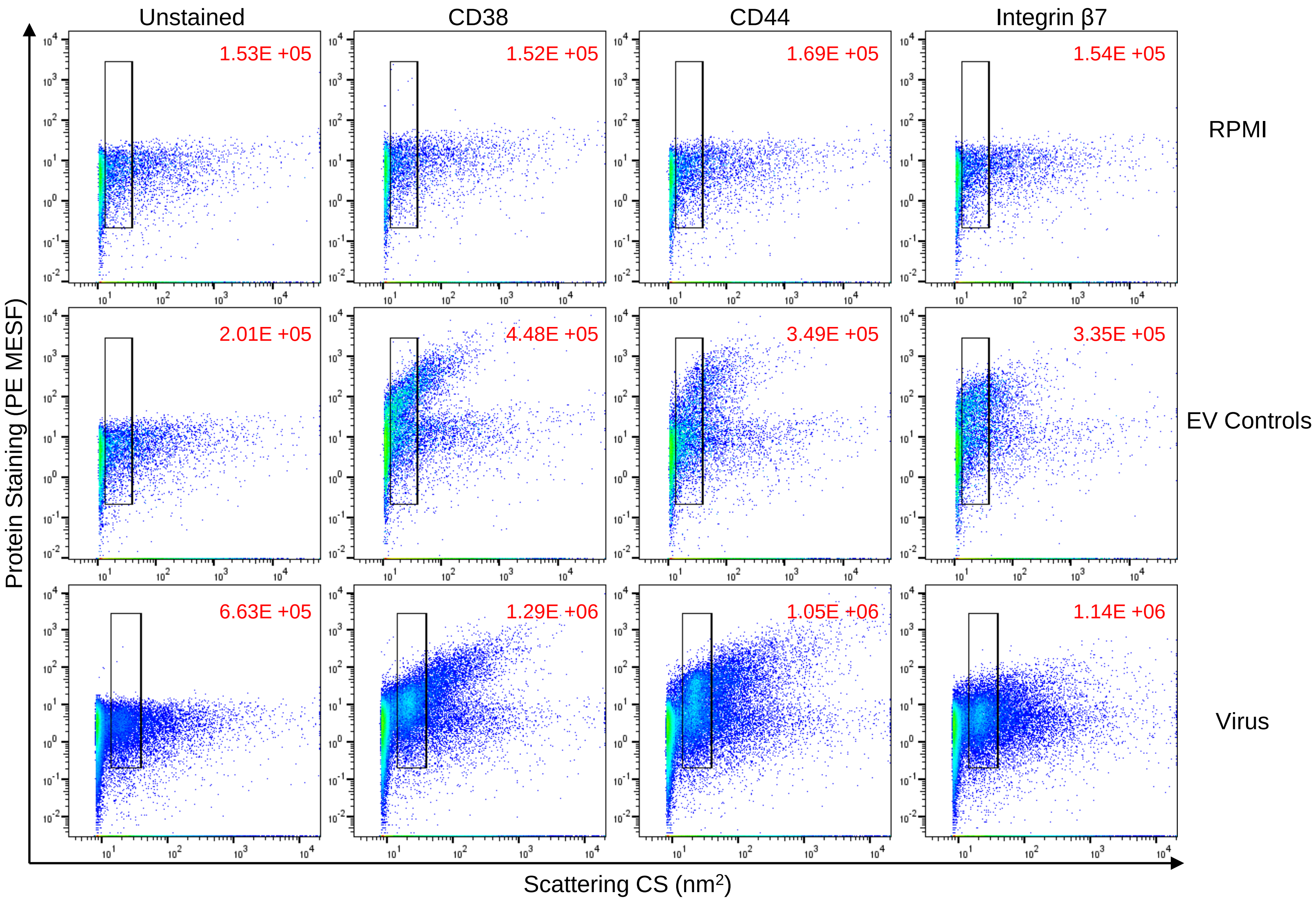

Fig. S3. Comparison of EV versus virus staining (related to Figure 5). Each row shows a set of stains performed on media alone (RPMI), cell culture supernatants from uninfected PBMC (EV control), and HIV_{BaL} virus from PBMC (Virus). Columns show staining with PE-labelled antibodies against CD38, CD44 and Integrin β7. Unstained samples are shown in column one as a control. Particle concentrations of the gated regions are shown in red on each dot plot.

FCMPass Plots

Figure 1 | Scatter Calibration Plots

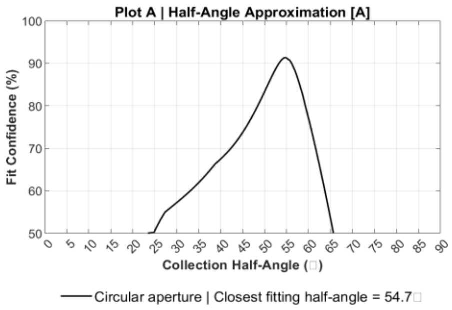

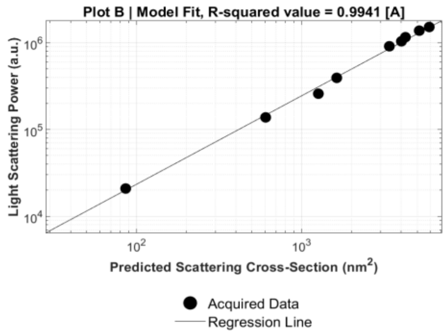

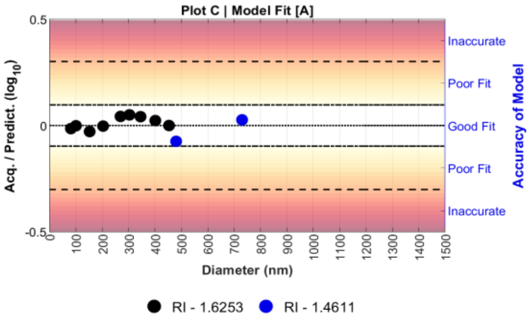

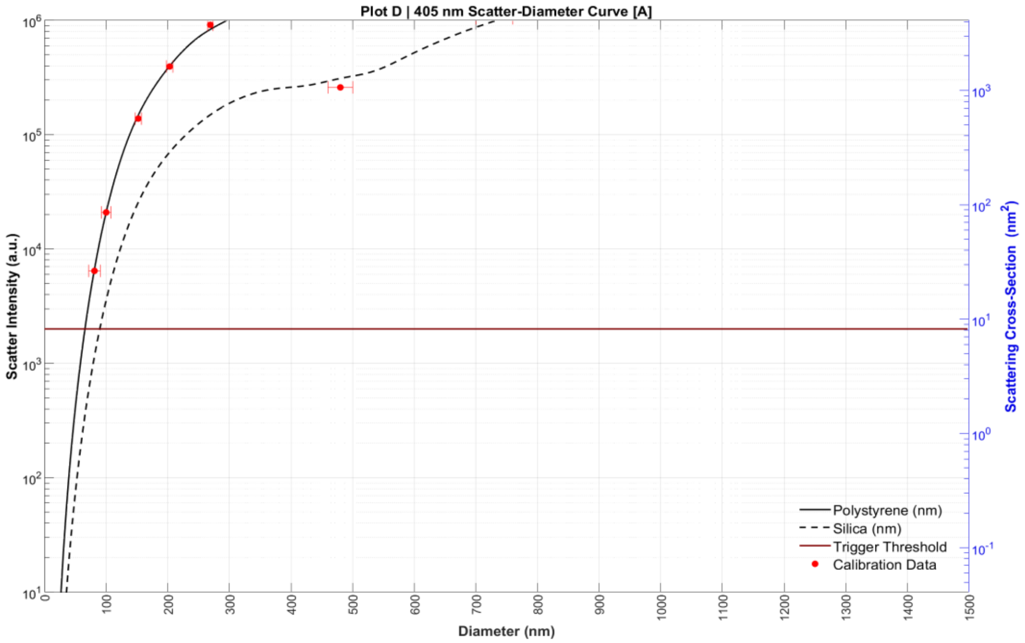

Figure 2 | Fluorescence Calibration Plots

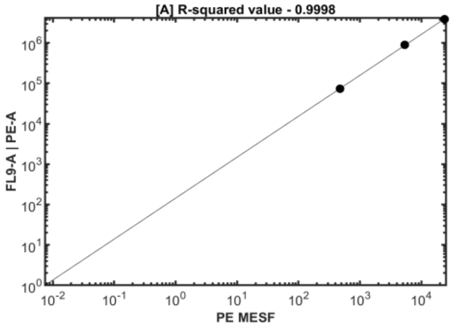

MIFlowCytEv Table

Framework Criteria	What to report	Please complete each criterion
1.1 Preanalytical variables conforming to MISEV guidelines.	Preanalytical variables relating to EV sample including source, collection, isolation, storage, and any others relevant and available in the performed study.	Virus containing cell supernatants were generated, collected, and stored as described in the Materials and Methods section. No further isolation techniques were used.
1.2 Experimental design according to MIFlowCyt guidelines.	EV-FC manuscripts should provide a brief description of the experimental aim, keywords, and variables for the performed FC experiment(s) using MIFlowCyt checklist criteria: 1.1, 1.2, and 1.3, respectively. Template found at www.evflowcytometry.org.	
2.1 Sample staining details	State any steps relating to the staining of samples. Along with the method used for staining, provide relevant reagent descriptions as listed in MIFlowCyt guidelines (Section 2.4 Fluorescence Reagent(s) Descriptions).	Virus samples were stained with PE conjugated antibodies against cellular proteins. Methods used for staining are as described in in the Materials and Methods.
2.2 Sample washing details	State any steps relating to the washing of samples.	Virus samples were not washed.
2.3 Sample dilution details	All methods and steps relating to sample dilution.	Unlabelled virus supernatants were serially diluted prior to acquisition on the flow cytometer to estimate virus particle concentration. Antibody labeled virus samples were diluted 1:250-1:1000 with PBS prior to acquisition on the flow cytometer. See methods in the manuscript for detailed description.
3.1 Buffer alone controls.	State whether a buffer-only control was analyzed at the same settings and during the same experiment as the samples of interest. If utilized it is recommended that all samples be recorded for a consistent set period of time e.g. 5 minutes, rather than stopping analysis at a set recorded event count e.g. 100,000 events. This allows comparisons of total particle counts between controls and samples.	Media only sample controls were run (Fig. S3).
3.2 Buffer with reagent controls.	State whether a buffer with reagent control was analyzed at the same settings, same concentrations, and during the same experiment as the samples of interest. If used state what the results were.	Media containing antibody controls were run for each individual antibody used in Figure 5.
3.3 Unstained controls.	State whether unstained control samples were analyzed at the same settings and during the same experiment as stained samples. If used, state what the results were, preferably in standard units.	Unstained virus samples were analysed using the same settings as stained samples (Fig. S3).
3.4 Isotype controls.	The use of isotype controls is applicable to immunofluorescence labelling only. State whether isotype controls were analyzed at the same settings and during the same experiment as stained samples. If utilized, state which antibody they are matched to, the concentration used, and what the results were (Section 4.2, 4.3, 4.4). Due to conjugation differences between manufacturers if should be stated if the isotype controls are from the same manufacturer as the matched antibodies.	Isotype controls were acquired at the same settings as target antibodies. More information about the 10 isotype controls that were used and the antibodies that they are matched to can be found within the LEGENDScreen user manual.
3.5 Single-stained controls.	State whether single-stained controls were included. If used state whether the single-stained controls were recorded using the same settings, dilutions, and during the same experiment as stained samples and state what the results were, preferably in standard units (Section 4.2, 4.3, 4.4).	Single stained controls were included on cell culture media, recorded in the same settings, and dilutions. See Figures S3 for results showing single stained controls in standard units of MESF.
3.6 Procedural controls.	State whether procedural controls were included. If used, state the procedure and if the procedural controls were acquired at the same settings and during the same experiment as stained samples.	Media controls containing no virus were included, recorded with the same settings, and dilutions. See Figures S3, for results showing procedural Procedural controls in standard units of MESF.
3.7 Serial dilutions.	State whether serial dilutions were performed on samples and note the dilution range and manner of testing. The fluorescence and/or scatter signal intensity would ideally be reported in standard units (see Section 4.3, 4.4) but arbitrary units can also be used. This data is best reported by plotting the recorded number events/concentration over a set period of time at different sample dilution. The median fluorescence intensity at each of the dilutions should also ideally be plotted on the same or a separate plot.	Serial dilutions were acquired and representative plots are displayed in Figure S2.
3.8. Detergent treated EV-samples	State whether samples were detergent treated to assess lability. If utilized, state what detergent was used, the end concentration of the detergent, and what the results were of the lysis.	NA - these were not EV samples
4.1 Trigger Channel(s) and Threshold(s).	The trigger channel(s) and threshold(s) used for event detection. Preferably, the fluorescence calibration (Section 4.3) and/or scatter calibration (Section 4.4) should be used in order to report the trigger channel(s) and threshold(s) in standardized units.	Trigger Channel(s) and Threshold(s) in calibrated and arbitrary units are summarized for each sample in the table on the 'Sample Acq Sheet'.
4.2 Flow Rate / Volumetric quantification.	State if the flow rate was quantified/validated and if so, report the result and how they were obtained.	Flow Rate Volumetric quantification was performed using the application in the Beckman Coulter CytExperts acquisition software.
4.3 Fluorescence Calibration.	State whether fluorescence calibration was implemented, and if so, report the materials and methods used, catalogue numbers, lot numbers, and supplied reference units for the standards. Fluorescence parameters may be reported in standardized units of MESF, ERF, or ABC beads. The type of regression used, and the resulting scatter plot of arbitrary data vs standard data for the reference particles should be supplied.	Fluorescence calibration was performed on PE using FCMPASS software as previously demonstrated [1,2]. Details on the calibration reagents and regression can be found in the summary table on the 'Fl Cali' sheet and in herein (FCMPass plots).
4.4 Light Scatter Calibration.	State whether and how light scatter calibration was implemented. Light scatter parameters may be reported in standardized units of nm2, along with information required to reproduce the model.	Light scatter calibration was performed using FCMPASS software as previously demonstrated [1,2]. Details on the reagents and modelling parameters can be found herein
5.1 EV diameter/surface area/volume approximation.	State whether and how EV diameter, surface area, and/or volume has been calculated using FC measurements.	Diameter approximation was not performed as FCMpass did not have the RI values for virus. Arbitrary units of light scatter were reported as scattering cross section (nm^2)
5.2 EV refractive index approximation.	State whether the EV refractive index has been approximated and how this was done.	NA- these are not EVs, refractive index approximation was not performed.
5.3 EV epitope number approximation.	State whether EV epitope number has been approximated, and if so, how it was approximated.	Epitope approximation was not specifically addressed, however fluorescence data was reported in PE MESF which has a approximated equivalence of 1 epitope to 1 PE MESF.
6.1 Completion of MIFlowCyt checklist.	Complete MIFlowCyt checklist criteria 1 to 4 using the MIFlowCyt guidelines. Template found at www.evflowcytometry.org.	Completion of MIFlowCyt checklist were included.
6.2 Calibrated channel detection range	If fluorescence or scatter calibration has been carried out, authors should state whether the upper and lower limits of a calibrated detection channel were calculated in standardized units. This can be done by converting the arbitrary unit scale to a calibrated scaled, as discussed in Section 4.3 and 4.4, and providing the highest unit on this scale and the lowest detectable unit above the unstained population. The lowest unit at which a population is deemed 'positive' can be determined a variety of ways, including reporting the 99th percentile measurement unit of the unstained population for fluorescence. The chosen method for determining at what unit an event was deemed positive should be clearly outlined.	Refer to data presented in FCMPAss Plots in this excel sheet for calibrated detection channel ranges.
6.3 EV number/concentration.	State whether EV number/concentration has been reported. If calculated, it is preferable to report EV number/concentration in a standardized manner, stating the number/concentration between a set detection range.	Virus particle concentrations of virus containing supernatants were reported for the gated regions denoted (identified by SSC) in Figure S3.
6.4 EV brightness.	When applicable, state the method by which the brightness of EVs is reported in standardized units of scatter and/or fluorescence.	Brightness as reported in MESF for fluorescence antibody labeling of cellular proteins was identified in Fig 5, and scattering cross section for light scatter.
7.1. Sharing of data to a public repository.	Provide a link to the experimental data in a public data repository.	Data has been shared on flowrepository.org in manuscript data availability section

Sample Acquisition Information

Filename	Sample Type	Trigger Parameter	Trigger ID	Trigger Threshold (au)	Detector Setting (au)	Trigger Threshold | Polystyrene (nm)	Trigger Threshold | Silica (nm)	Trigger Threshold | Scattering CS (nm^2)
All experimental samples	Sample	Violet SSC	SSC_1-H	2000	200	65.5	89.6	8.2
100 nm.fcs	SSC Calibrator	Violet SSC	SSC_1-H	2000	200	65.5	89.6	8.2
150 nm.fcs	SSC Calibrator	Violet SSC	SSC_1-H	2000	200	65.5	89.6	8.2
200 nm.fcs	SSC Calibrator	Violet SSC	SSC_1-H	2000	200	65.5	89.6	8.2
269 nm.fcs	SSC Calibrator	Violet SSC	SSC_1-H	2000	200	65.5	89.6	8.2
300 nm.fcs	SSC Calibrator	Violet SSC	SSC_1-H	2000	200	65.5	89.6	8.2
350 nm.fcs	SSC Calibrator	Violet SSC	SSC_1-H	2000	200	65.5	89.6	8.2
400 nm.fcs	SSC Calibrator	Violet SSC	SSC_1-H	2000	200	65.5	89.6	8.2
450 nm.fcs	SSC Calibrator	Violet SSC	SSC_1-H	2000	200	65.5	89.6	8.2
500 si.fcs	SSC Calibrator	Violet SSC	SSC_1-H	2000	200	65.5	89.6	8.2
700 si1.fcs	SSC Calibrator	Violet SSC	SSC_1-H	2000	200	65.5	89.6	8.2
80 nm.fcs	SSC Calibrator	Violet SSC	SSC_1-H	2000	200	65.5	89.6	8.2
MESF Beads	FL Calibrator	Violet SSC	SSC_1-H	2000	200	65.5	89.6	8.2

Fluorescence Calibration

Parameter	New Parameter Name	Reference Fluor	Figure No.	Ref.Value 1	Ref.Value 2	Ref.Value 3	Acq. Value 1	Acq. Value 2	Acq. Value 3	F/P Ratio	Transformation	Slope	Intercept	R-Square	Regression Type	Manufacturer	Cat. No.	Lot No.
FL9-A | PE-A	PE MESF	PE	2A	474	5359	23843	73309	896000	3870000	1	N/A	1.01	2.15649	0.99981	weighted log	BD	340495	47973

Scatter Calibration

SSC Parameter	Wavelength	Sheath RI	Determine HA	Aperture Geom	Theta	Phi	Eps	Cali. Factor (nm^2)
SSC_1-H | Violet SSC-H	405	1.343091653	on	circle	90	90	54.7	0.004105378

Light scatter calibration reference beads

Diameter (nm)	Diameter CV (%)	Measured RI	Measurement Wavelength	Composition	Acquired Stat	Acquisition Wavelength	Modelled RI	Manufacturer	Catalogue No.	Lot No.
81	11.7	1.59	589	Polystyrene	6435	405	1.625265271	ThermoFisher Scientific	3080A	228748
100	7.8	1.59	589	Polystyrene	20889		1.625265271		3100A	204935
152	3.3	1.59	589	Polystyrene	138043		1.625265271		3150A	202026
203	2.6	1.59	589	Polystyrene	394220		1.625265271		3200A	205131
269	1.6	1.59	589	Polystyrene	912000		1.625265271		3269	202729
303	1.6	1.59	589	Polystyrene	1160000		1.625265271		3300A	204665
345	1.9	1.59	589	Polystyrene	1380000		1.625265271		3350A	199283
401	1.3	1.59	589	Polystyrene	1520000		1.625265271		3400A	203859
453	1.7	1.59	589	Polystyrene	1800000		1.625265271		3450A	204047
480	4.2	1.45	589	Silica	258839		1.46110339		8050	203277
730	4.1	1.45	589	Silica	1040000		1.46110339		8070	207434

Instrument Settings

Files	Parameter	Gain (au)								
	FSC-H	FSC-A	SSC-H	SSC-A	SSC_1-H	SSC_1-A	FL6-H	FL6-A	FL9-H	FL9-A
All samples	73	73	53	53	200	200	1	1	3000	3000
